# Dietary Intake of Advanced Glycation End Products (AGEs) and Mortality among Individuals with Colorectal Cancer

**DOI:** 10.3390/nu13124435

**Published:** 2021-12-10

**Authors:** Ziling Mao, Elom K. Aglago, Zhiwei Zhao, Casper Schalkwijk, Li Jiao, Heinz Freisling, Elisabete Weiderpass, David J. Hughes, Anne Kirstine Eriksen, Anne Tjønneland, Gianluca Severi, Joseph Rothwell, Marie-Christine Boutron-Ruault, Verena Katzke, Rudolf Kaaks, Matthias B. Schulze, Anna Birukov, Vittorio Krogh, Salvatore Panico, Rosario Tumino, Fulvio Ricceri, H. Bas Bueno-de-Mesquita, Roel C. H. Vermeulen, Inger T. Gram, Guri Skeie, Torkjel M. Sandanger, J. Ramón Quirós, Marta Crous-Bou, Maria-Jose Sánchez, Pilar Amiano, María-Dolores Chirlaque, Aurelio Barricarte Gurrea, Jonas Manjer, Ingegerd Johansson, Aurora Perez-Cornago, Mazda Jenab, Veronika Fedirko

**Affiliations:** 1 Department of Epidemiology, Rollins School of Public Health, Emory University, Atlanta, GA 30322, USA; zim12@pitt.edu (Z.M.); zhiwei.zhaoxmu@hotmail.com (Z.Z.); 2 Department of Epidemiology, Graduate School of Public Health, University of Pittsburgh, Pittsburgh, PA 15261, USA; 3 Section of Nutrition and Metabolism, International Agency for Research on Cancer, World Health Organization (IARC-WHO), 69372 Lyon, France; AglagoE@fellows.iarc.fr (E.K.A.); Freislingh@iarc.fr (H.F.); Director@iarc.fr (E.W.); jenabm@iarc.fr (M.J.); 4 Department of Internal Medicine, School for Cardiovascular Diseases (CARIM), Maastricht University, 6229ER Maastricht, The Netherlands; c.schalkwijk@maastrichtuniversity.nl; 5 Baylor College of Medicine, 2002 Holcombe Blvd, Houston, TX 77030, USA; jiao@bcm.edu; 6 Cancer Biology and Therapeutics Group, School of Biomolecular and Biomedical Science, UCD Conway Institute, University College Dublin, Belfield, D04 V1W8 Dublin, Ireland; david.hughes@ucd.ie; 7 Danish Cancer Society Research Center, Diet, Genes and Environment Nutrition and Biomarkers (NAB), Strandboulevarden 49, DK-2100 Copenhagen, Denmark; ake@cancer.dk (A.K.E.); annet@cancer.dk (A.T.); 8 CESP (UMR1018), Faculté de Médecine Université Paris-Saclay, Inserm, Gustave Roussy, 94805 Villejuif, France; gianluca.severi@inserm.fr (G.S.); joseph.rothwell@gustaveroussy.fr (J.R.); marie-christine.boutron@gustaveroussy.fr (M.-C.B.-R.); 9 Department of Statistics, Computer Science and Applications (DISIA), University of Florence, 50121 Florence, Italy; 10 Division of Cancer Epidemiology, German Cancer Research Center (DKFZ), 69120 Heidelberg, Germany; V.Katzke@Dkfz-Heidelberg.de (V.K.); r.kaaks@Dkfz-Heidelberg.de (R.K.); 11 Department of Molecular Epidemiology, German Institute of Human Nutrition Potsdam-Rehbruecke, 14558 Nuthetal, Germany; mschulze@dife.de (M.B.S.); Anna.Birukov@dife.de (A.B.); 12 Institute of Nutritional Science, University of Potsdam, 14558 Nuthetal, Germany; 13 Epidemiology and Prevention Unit, Fondazione IRCCS Istituto Nazionale dei Tumori di Milano, Via Venezian, 20133 Milan, Italy; vittorio.krogh@istitutotumori.mi.it; 14 Dipartmento Di Medicina Clinica E Chirurgia, Federico II University, 80131 Naples, Italy; salvatorepanico2@gmail.com; 15 Cancer Registry and Histopathology Department, Provincial Health Authority (ASP 7), 97100 Ragusa, Italy; rtuminomail@gmail.com; 16 Hyblean Association for Epidemiological Research, AIRE—ONLUS, 97100 Ragusa, Italy; 17 Department of Clinical and Biological Sciences, University of Turin, 10043 Turin, Italy; fulvio.ricceri@unito.it; 18 Centre for Nutrition, Prevention and Health Services, National Institute for Public Health and the Environment (RIVM), 3720 Bilthoven, The Netherlands; basbuenodemesquita@gmail.com; 19 Division of Environmental Epidemiology, Institute for Risk Assessment Sciences, Utrecht University, 80178 Utrecht, The Netherlands; R.C.H.Vermeulen@uu.nl; 20 Department of Community Medicine, University of Tromsø, The Arctic University of Norway, 9010 Tromsø, Norway; inger.gram@uit.no (I.T.G.); guri.skeie@uit.no (G.S.); torkjel.sandanger@uit.no (T.M.S.); 21 Public Health Directorate, 33080 Asturias, Spain; joseramon.quirosgarcia@asturias.org; 22 Unit of Nutrition and Cancer, Cancer Epidemiology Research Program, Catalan Institute of Oncology (ICO)—Bellvitge Biomedical Research Institute (IDIBELL), L’Hospitalet de Llobregat, 08908 Barcelona, Spain; marta.crous@iconcologia.net; 23 Department of Epidemiology, Harvard T.H. Chan School of Public Health, Boston, MA 02115, USA; 24 Escuela Andaluza de Salud Pública (EASP), 18011 Granada, Spain; mariajose.sanchez.easp@juntadeandalucia.es; 25 Instituto de Investigación Biosanitaria ibs.GRANADA, 18012 Granada, Spain; 26 Centro de Investigación Biomédica en Red de Epidemiología y Salud Pública (CIBERESP), 28029 Madrid, Spain; 27 Department of Preventive Medicine and Public Health, University of Granada, 18012 Granada, Spain; 28 Ministry of Health of the Basque Government, Sub Directorate for Public Health and Addictions of Gipuzkoa, 20013 San Sebastian, Spain; epicss-san@euskadi.eus; 29 Epidemiology of Chronic and Communicable Diseases Group, Biodonostia Health Research Institute, 20013 San Sebastian, Spain; 30 Spanish Consortium for Research on Epidemiology and Public Health (CIBERESP), Instituto de Salud Carlos III, 28029 Madrid, Spain; mdolores.chirlaque@carm.es (M.-D.C.); aurelio.barricarte.gurrea@navarra.es (A.B.G.); 31 Department of Epidemiology, Regional Health Council, IMIB-Arrixaca, Murcia University, 30100 Murcia, Spain; 32 Navarra Public Health Institute, 31003 Pamplona, Spain; 33 Navarra Institute for Health Research (IdiSNA), 31008 Pamplona, Spain; 34 Department of Surgery, Skåne University Hospital Malmö, Lund University, SE-221 00 Malmö, Sweden; jonas.manjer@med.lu.se; 35 Department of Epidemiology and Clinical Medicine, Umeå University, SE-901 87 Umeå, Sweden; ingegerd.johansson@umu.se; 36 Cancer Epidemiology Unit, Nuffield Department of Population Health, University of Oxford, Oxford OX3 7LF, UK; aurora.perez-cornago@ndph.ox.ac.uk; 37 MD Anderson Cancer Center, Department of Epidemiology, 1515 Holcombe Blvd., Unit 1340, Houston, TX 77030, USA

**Keywords:** advanced glycation end-products, dietary advanced glycation end-products, all-cause mortality, colorectal cancer mortality

## Abstract

Advanced glycation end-products (AGEs) may promote oxidative stress and inflammation and have been linked to multiple chronic diseases, including cancer. However, the association of AGEs with mortality after colorectal cancer (CRC) diagnosis has not been previously investigated. Multivariable Cox proportional hazards models were used to calculate hazard ratios and corresponding 95% confidence intervals for associations between dietary intake of AGEs with CRC-specific and all-cause mortality among 5801 participant cases diagnosed with CRC in the European Prospective Investigation into Cancer and Nutrition study between 1993 and 2013. Dietary intakes of AGEs were estimated using country-specific dietary questionnaires, linked to an AGE database, that accounted for food preparation and processing. During a median of 58 months of follow-up, 2421 cases died (1841 from CRC). Individually or combined, dietary intakes of AGEs were not associated with all-cause and CRC-specific mortality among cases. However, there was a suggestion for a positive association between AGEs and all-cause or CRC-specific mortality among CRC cases without type II diabetes (all-cause, *P_interaction_* = 0.05) and CRC cases with the longest follow-up between recruitment and cancer diagnosis (CRC-specific, *P_interaction_* = 0.003; all-cause, *P_interaction_* = 0.01). Our study suggests that pre-diagnostic dietary intakes of AGEs were not associated with CRC-specific or all-cause mortality among CRC patients. Further investigations using biomarkers of AGEs and stratifying by sex, diabetes status, and timing of exposure to AGEs are warranted.

## 1. Introduction

Colorectal cancer (CRC) is the third leading cause of cancer death worldwide [1]. Large differences in incidence rates across countries and rapid changes in incidence among migrant populations suggest that modifiable environmental factors, including diet and lifestyle, play an important role in CRC etiology [2]. Despite advances in screening and treatment, there is an increasing number of CRC survivors that are at risk for CRC recurrence and death [3]. Multiple lifestyle and dietary factors such as tobacco smoking, obesity, and heavy alcohol intake have been linked to the risk of CRC [4]. However, knowledge of the risk factors affecting survival after CRC diagnosis is limited. It is of interest to determine whether modifiable factors may affect cancer survival, particularly those that could be modified by changes in diet or lifestyle.

Advanced glycation end products (AGEs) comprise a large heterogeneous group of compounds derived from a series of irreversible reactions between reducing sugars and free amino groups in amino acids, or oxidation of sugars, lipids, and amino acids [5]. Endogenous AGEs are formed in the human body under physiological conditions, and their formation is enhanced in people with diabetes [6]. A major exogenous source of the pool of AGEs in our body is from the diet [7,8]. Foods rich in both fat and protein and cooked at high temperature and with dry heat processing, such as grilling, broiling, frying, or roasting, tend to be the major sources of dietary AGEs [9]. The best characterized dietary AGEs include N^ε^-[carboxymethyl]lysine (CML), N^ε^-[1-carboxyethyl]lysine (CEL), and N^δ^-[5-hydro-5-methyl-4-imidazolon-2-yl]-ornithine (MG-H1) [10].

Experimental studies have suggested that the accumulation of AGEs can increase oxidative stress and inflammation in the tissue through binding to the receptor for advanced glycation end products (RAGEs) [11,12,13,14,15,16]. Another mechanism through which AGEs induce pathological effects is via the cross-linking of collagen and other proteins, which could contribute to structural and physiologic changes in the cardiovascular system [17,18]. Through the above mechanisms, AGEs may act as a risk factor for aging-related health outcomes [11,12,13], including reduced survival among cancer patients. In epidemiologic studies, higher AGEs have been associated with various chronic diseases, including some cancers and cardiovascular disease (CVD) [19,20,21,22,23,24], which are associated with reduced survival. However, the results of previously published observational studies on the association of AGEs with mortality risk were inconsistent [21,25,26,27,28,29,30,31], which in part could be due to different populations included in the studies (e.g., patients with diabetes or breast cancer, or older healthy individuals) and different methods for estimating exposure to AGEs. Among these, most studies focused on AGE measurements in plasma [21,26,27,28,29,30], and only two prospective studies [25,31] investigated the associations of dietary AGEs with mortality risk. The first [25] reported a positive association between higher post-diagnosis dietary AGE intake and all-cause, CVD, and breast cancer mortality among breast cancer patients. However, the second study among healthy Japanese adults [31] suggested that dietary AGEs are not associated with higher risk of all-cause mortality. To our knowledge, so far, no prospective studies have investigated the association of dietary AGEs with mortality risk among CRC patients. 

Therefore, we examined the associations between pre-diagnostic dietary intakes of the three best characterized AGEs—CML, CEL, and MG-H1—and CRC-specific and all-cause mortality among individuals diagnosed with CRC in the large, multi-center prospective cohort, the European Prospective Investigation into Cancer and Nutrition (EPIC) study. We hypothesized that higher dietary intakes of these AGEs before cancer diagnosis are associated with higher mortality risk.

## 2. Methods

### 2.1. Study Population and Data Collection

The EPIC is a large, multi-center prospective cohort study with more than 520,000 participants. The details and methods of the EPIC study have been reported previously [32,33]. Participating countries include France, Germany, Greece, Italy, The Netherlands, Spain, the United Kingdom, Sweden, Denmark, and Norway. Between 1992 and 1998, standardized lifestyle/personal history questionnaires, anthropometric data, and blood samples were collected from most participants at recruitment, before disease onset or diagnosis. 

Individuals who were eligible for the study were recruited from the general population of a specific geographical area, town, or province. Exceptions included the French sub-cohort and the Utrecht (The Netherlands) sub-cohort: the former is based on members of the health insurance system or state-school employees, while the latter is based on women who underwent screening for breast cancer. In addition, a portion of the Spanish and Italian sub-cohorts included blood donors. In our analysis, we included participants from all centers except Greece (excluded due to data restriction issues). Lifestyle questionnaires were used to obtain information on education, physical activity, lifetime alcohol intake, smoking status, and self-reported diabetes mellitus status at baseline. Anthropometric measures were assessed at recruitment, and body mass index (BMI) was computed as weight in kilograms over height in square meters.

The EPIC study was approved by the Ethical Review Board of the International Agency for Research on Cancer (IARC) and the Institutional Review Boards of each participating center. Written consent was obtained from all EPIC participants upon enrollment into the study.

### 2.2. Dietary Assessment and Estimation of AGE Intake

In EPIC, country- or center-specific validated dietary questionnaires (DQs) were completed at baseline, accounting for the usual food intake during the previous 12 months [32]. The Netherlands, Germany, Northern Italy, and France used quantitative DQs. In Spain and Ragusa (Italy), the quantitative DQs were interviewer-administered and structured by meals. Malmö (Sweden) and the UK used semi-quantitative food frequency questionnaires in combination with 7-day and 14-day records, respectively. In Umeå (Sweden), Denmark, Norway, and Naples (Italy), semi-quantitative food frequency questionnaires (FFQs) were used. Harmonization of food groups and portion sizes for quantification was carried out centrally at the IARC [34]. Cooking methods were not included in the DQ and FFQ. We used the most common cooking methods in a given country reported in the 24-hour recalls either in the EPIC calibration study or in national surveys.

Dietary AGEs were estimated using a reference dietary AGE food composition database, which is based on the CML, CEL, and MG-H1 concentrations (in mg/100 g of food) obtained from 190 food items commonly consumed in Europe using ultra-performance liquid chromatography tandem mass-spectrometry analysis [10]. Foods from the reference database were matched to those included in the DQs by name and descriptors, particularly those pertaining to preparation and processing whenever applicable [35]. Generic or multi-ingredient DQ foods were decomposed into more specific foods or ingredients based on country-specific recipes obtained from previous EPIC projects [34,36]. The EPIC-specific AGE composition database was then generated and used to obtain the daily intake (mg/day) of CML, CEL, and MG-H1 per study participant. The validity of these data was further confirmed by assessing the expected associations between higher dietary intakes of any of three AGEs and weight gain after an average of five years of follow-up in the same study population [36].

### 2.3. Cancer Ascertainment and Follow-Up

Cancer data was coded using the International Classification of Disease (ICD)-10 and the second revision of the ICD for Oncology (ICD-O-2). CRC cases included participants who developed colon (C18.0–C18.7), rectal (C19–C20), and overlapping or unspecified origin colorectal tumors (C18.8–C18.9). CRC included colon and rectal cancer cases. Colon cancer included tumors that developed in both the proximal site (C18.0–C18.5: cecum, appendix, ascending colon, hepatic flexure, transverse colon, and splenic flexure) and the distal site (C18.6–C18.7: descending and sigmoid colon).

Incident cancer cases were ascertained through record linkage with regional cancer registries (Denmark, Italy, The Netherlands, Norway, Spain, Sweden, and the United Kingdom; complete up to 2011–2013) or through a combination of methods, including the use of health insurance records, contacts with cancer and pathology registries, and active follow-up through study subjects and their next-of-kin (France and Germany; complete up to 2008 and 2009). Of 6027 identified CRC cases, we excluded individuals who were missing dietary AGE data (*n* = 99), had tumor stage coded as in situ (*n* = 3), had a follow-up time of zero due to cancer diagnosis listed on the death certificate (*n* = 4), or reported extreme total energy intakes (top and bottom 1% of the total energy intake to estimated energy requirements ratio; *n* = 120), leaving 5801 CRC cases for the final analytic cohort.

### 2.4. Vital Status Follow-Up

Vital status follow-up was determined through record linkage with regional and/or national mortality registries (Denmark, Italy, The Netherlands, Spain, and the United Kingdom) or active follow-up (France and Germany). Censoring dates for complete follow-up were between January 2013 and February 2015. Mortality was coded using ICD-10 (which includes Injuries and Causes of Death), and the outcome was assigned based on the underlying cause of death. 

### 2.5. Statistical Analyses

Dietary intakes of CML, CEL, and MG-H1 were natural log (ln)-transformed, and total energy intake was adjusted using the residual method [37]. For energy adjustment, we computed standardized residuals of each of the three AGEs by regressing the ln-transformed AGEs on total energy intake, sex, and center and adding back the sex- and center-specific mean to each observation. The combined AGE value was calculated as the sum of the three total energy-adjusted AGEs. The total energy-adjusted AGEs were analyzed separately and combined on a continuous scale per standard deviation (SD) increment and as quintiles of intake across all centers. 

Death from CRC was the primary endpoint, and death from any cause was used as a secondary endpoint. Entry time was age at first tumor diagnosis, and exit time was either death or censoring date (lost to or end of follow-up), whichever event occurred first. Cox proportional hazard models were used to calculate hazard ratios (HRs) and 95% confidence intervals (CIs). The proportional hazard assumptions for all variables in the model were tested with Schoenfeld residuals and included a time-dependent covariate in the Cox model. Two main models were fitted with different sets of adjustments. Model 1 was stratified by center and adjusted for sex, age at diagnosis (continuous, years), tumor stage (I, II, III, IV, missing), and total energy intake (continuous, kcal/day). To determine the final model (Model 2), the following a priori identified covariates were assessed as potential confounders: grade of tumor differentiation (well, moderately, poorly differentiated, unknown), location of primary tumor (colon or rectum), smoking status (never smoker, former smoker, current smoker, unknown), BMI (kg/m^2^), year of diagnosis, dietary intakes (red and processed meats, fruits and vegetables, dietary calcium, dietary fiber, sugar, dairy, alcohol drinking pattern), physical activity, and type II diabetes based on self-reporting at baseline and ascertainment before cancer diagnosis. These variables were chosen based on previous published evidence showing their associations with CRC incidence or survival and/or AGEs. We evaluated confounding by assessing change (>10%) in HRs after including the variables in the model. The final Model 2 was stratified by center and adjusted for year of diagnosis (continuous), location of tumor (colon/rectum), BMI (continuous, kg/m^2^), smoking status (never, former, current, missing), and type II diabetes (no, yes, missing; defined as being diagnosed with diabetes at baseline or during follow-up). Participants with missing values were included in all analyses, and respective variables were coded with a missing value indicator, unless otherwise specified. *p*-value for trend was calculated, with the median value of each AGE quintile included as a continuous variable in the corresponding models. 

To evaluate the linearity of the dose-response associations between continuous intakes of AGEs and risk for CRC-specific and all-cause mortality, non-parametric restricted cubic splines [38,39] were fitted to a Cox proportional hazard model using the SAS macro “lgtphcurv9” [40]. Tests for non-linearity used the likelihood ratio test, comparing the model with only the linear term to the model with both the linear and cubic spline terms [40]. 

We assessed whether missing tumor stage information influenced the effect estimates using several approaches. The first approach reclassified missing tumor stage values into a separate missing category and adjusted for the stage variable in the final model (included in the primary analysis). Second, a sensitivity analysis was conducted by excluding participants with missing stage information and subsequently by assessing how the results were affected by the missing stage information. Finally, an imputation of missing tumor stage values was conducted using the SAS PROC MI procedure as described previously [41]. The multiple imputation method was based on available data for the other covariates in the model and assumed that the stage data was missing at random.

Stratified analyses by categories of potentially biologically relevant effect modifiers (time interval between recruitment and CRC diagnosis, length of follow-up, sex, age at diagnosis, tumor site, grade, and stage, BMI, physical activity, smoking status, alcohol intake, and prevalent and incident diabetes) were conducted. Stratified multivariable-adjusted HRs and 95% CIs were reported per 1 SD increase in CML, CEL, MG-H1, and combined AGEs. A cross-product of AGE as a continuous variable and the covariate of interest as a continuous or categorical variable was included in the model to test for multiplicative statistical interaction; the likelihood ratios based on the models with and without the interaction terms were used to test for statistical significance.

All statistical tests were conducted using SAS version 9.2 (SAS Institute, Cary, NC, USA). *p*-values of <0.05 were considered statistically significant.

## 3. Results

### 3.1. Patient Characteristics

Among the 5801 eligible CRC cases, over a mean of 68 (median = 58) months of follow up, 2421 died of any cause (including 1841 from CRC). Selected baseline characteristics of study participants across quintiles of the combined AGEs are shown in Table 1. CRC cases in the highest compared to the lowest quintile of the combined AGEs were less likely to be current smokers and, on average, had lower red meat consumption.

### 3.2. Dietary Intakes of AGEs and Mortality among CRC Patients

The associations of CML, CEL, MG-H1, and the combined AGEs with CRC-specific and all-cause mortality are shown in Table 2. In our study population, the pre-diagnostic dietary intakes of AGEs were not statistically significantly associated with CRC-specific or all-cause mortality risk. For CRC-specific mortality, the fully adjusted HRs for the highest relative to the lowest quintile (HR_Q5_ vs. _Q1_) of CML, CEL, MG-H1, and the combined AGEs were 1.16 (95% CI: 0.98–1.36, *P_trend_* = 0.23), 1.11 (95% CI: 0.94–1.31, *P_trend_* = 0.13), 1.10 (95% CI: 0.94–1.28, *P_trend_* = 0.24), and 1.09 (95% CI: 0.93–1.28, *P_trend_* = 0.29), respectively. For all-cause mortality, the fully adjusted HR_Q5_ vs. _Q1_ of CML, CEL, MG-H1, and the combined AGEs were 1.13 (95% CI: 0.98–1.30, *P_trend_* = 0.36), 1.03 (95% CI: 0.89–1.19, *P_trend_* = 0.47), 1.09 (95% CI: 0.95–1.25, *P_trend_* = 0.23), and 1.08 (95% CI: 0.94–1.24, *P_trend_* = 0.33), respectively. Although the suggestive positive associations for MG-H1 and combined AGEs were observed mostly among women, no statistically significant interactions by sex were identified (Supplemental Table S1). *p*-values for nonlinearity tests from the restricted cubic splines models were generally consistent with a linear response (Supplemental Figures S1 and S2), except for CML, which were consistent with a non-linear response. 

### 3.3. Sensitivity Analyses 

Excluding participants with missing stage information (around 26%) or using imputed missing tumor stage data had no effect on the results (Supplemental Table S2) We also assessed whether the association between dietary AGEs and risk of mortality differed for long-term survivors. After the exclusion of cases that occurred during the first 5 years of follow-up, a positive association of pre-diagnostic dietary AGEs with CRC-specific mortality was found for CML (HR = 1.19, 95% CI: 1.04–1.36; *P_trend_* = *0.01*) and CEL (HR = 1.15, 95% CI: 1.02–1.30; *P_trend_* = *0.02*) (Supplemental Table S2). Similar results were also observed for all-cause mortality (Supplemental Table S3). 

### 3.4. Stratified Analyses

**Figure 1:**
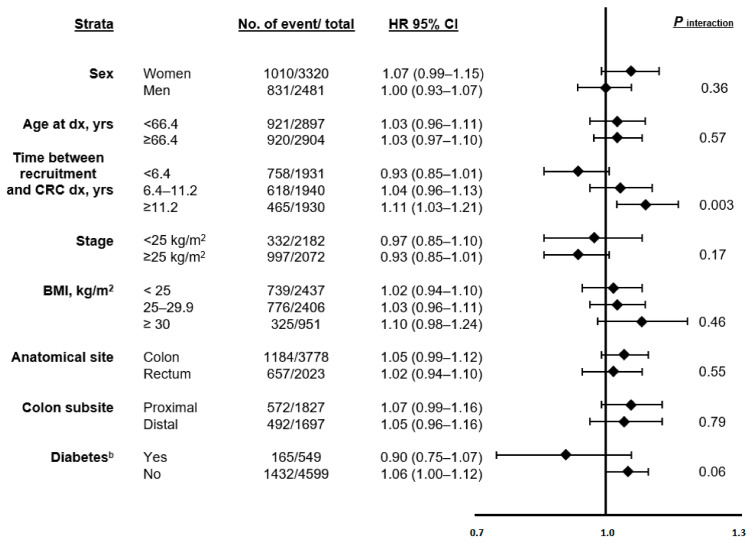
The associations of pre-diagnostic combined dietary intake of AGEs ^a^ with CRC-specific mortality across strata of selected patient characteristics among individuals with CRC in the EPIC study (*n* = 5801). Abbreviations: AGE, advanced glycation end products; CRC, colorectal cancer. ^a^ Combined AGEs: CML+CEL+MGH1. ^b^ Diabetes based on self-reporting at baseline and ascertainment during follow-up.

Stratified analyses suggested differences in the associations between dietary AGEs and CRC-specific (Figure 1 and Supplemental Table S2) and all-cause mortality (Supplemental Table S3) across select subcategories of potential a priori defined biologically plausible effect modifiers. Statistically significant positive associations between AGEs and mortality risk were observed among participants who were diagnosed with CRC more than 11 years after recruitment (CRC-specific mortality, per one SD change in AGE; HR _CML_ = 1.09, 95% CI: 1.00–1.20; HR _CEL_ = 1.11, 95% CI: 1.02–1.20, HR _MG-H1_ = 1.11, 95% CI: 1.02–1.21, and HR _AGEs_ = 1.11, 95% CI: 1.03–1.21; all *P_interaction_* < *0.01*). There were also some indications that the associations of CML, CEL, MG-H1, and the combined AGEs were slightly stronger among participants without type II diabetes, with HRs of 1.07 (95% CI: 1.01–1.13), 1.05 (95% CI: 1.00–1.12), 1.06 (95% CI: 1.00–1.12), and 1.06 (95% CI: 1.00–1.12) for CRC-specific mortality, and 1.05 (95% CI: 1.00–1.11), 1.04 (95% CI: 1.00–1.09), 1.05 (95% CI: 1.00–1.10), and 1.05 (95% CI: 1.00–1.11) for all-cause mortality, respectively. 

## 4. Discussion

Our findings suggest that pre-diagnostic dietary intakes of AGEs were not associated with CRC-specific and all-cause mortality among CRC patients in this large prospective study. Stratified analyses suggested potential interactions by time between recruitment and CRC diagnosis and that the association might be limited to individuals without type II diabetes and with more than 5 years of follow-up after cancer diagnosis. 

AGEs are associated with oxidative stress and inflammation [5,9], which can be involved in the initiation and progression of multiple chronic diseases, including cancers [11,12,13]. The accumulation of AGEs can activate intracellular signals via binding to RAGEs, which in turn can promote inflammation and tissue injury sustained by a RAGE-dependent expression of proinflammatory mediators, such as circulating monocyte chemotactic protein 1 (MCP-1) and vascular cell adhesion molecule 1 (VCAM-1) [5]. Exposure to AGEs can also lead to increased levels of reactive oxygen species, which are associated with an increase in oxidative stress. Both inflammation and oxidative stress are associated with cellular and DNA damage, which could lead to carcinogenic mutation and subsequent initiation, development, and progression of CRC [42,43,44]. In addition, AGEs might crosslink with the proteins, leading to functional alterations of the vasculature and angiogenesis [17,18], which might contribute to carcinogenesis [45]. AGEs could also be associated with adverse outcomes, including recurrence and death, among CRC survivors. Evidence from previous clinical trials has linked an AGE-restricted diet to a decrease in plasma AGEs and to markers of oxidative stress and inflammation [13,15,46], which might support the idea that dietary AGEs contribute to the pool of AGEs in the human body and could lead to CRC development and progression. 

Only two previous epidemiologic studies investigated the association of dietary AGE intakes with all-cause or cause-specific mortality risk and were limited to only dietary CML [25,31]. A prospective study among postmenopausal women diagnosed with invasive breast cancer (*n* = 2073) in the Women’s Health Initiative (WHI) study reported a statistically significant positive association between higher post-diagnosis dietary intake of CML and an increased risk of all-cause (HR = 1.51, 95% CI: 1.17–1.94), CVD (HR = 1.86, 95% CI: 1.19–2.91), and breast cancer (HR = 2.14, 95% CI: 1.19–3.84) mortality [25]. However, the investigators measured CML only, and the assessment of dietary CML was based on food composition as determined via an enzyme-linked immunosorbent assay (ELISA) method, which is different from our quantitative instrumental mass-spectrometry method for assessing dietary AGEs. The other study in a Japanese prospective cohort (men = 13,335 and women = 15,724) suggested that dietary CML intake estimated using a reference liquid chromatography tandem mass-spectrometry-based data set was not statistically significantly associated with higher mortality in healthy adults [31]. However, among men, higher CML intake was associated with lower risk for all-cause mortality (HR = 0.89, 95% CI: 0.79–1.00; *P*_trend_ = *0.05*). Considering differences in timing of exposure (after cancer diagnosis in the WHI study), AGE assessment methods (ELISA), and study populations (breast cancer survivors and healthy adults), our results among patients with CRC may not be directly comparable with previous findings. However, somewhat consistent with previous findings, our study suggested that the positive AGE-mortality association might be stronger among women. A prospective cohort study from Finland of non-diabetic men (*n* = 535) and women (*n* = 606) also reported a stronger positive association between AGE-modified bovine serum albumin (BSA), measured via ELISA, and all-cause mortality in women but not men [30]. The reason behind this difference is not clear, but it is possible that the biological differences between men and women in metabolism and immune response could account for both our and previous findings. Earlier evidence suggested that AGE accumulation is associated with positive expression of estrogen receptor alpha among breast cancer patients and supported a potential mechanistic link between AGEs and estrogen signaling [47,48]. This potential mechanism might contribute to the stronger AGE-mortality associations in women with CRC, although we did not observe any differences in women by menopausal status. 

In our sensitivity analyses, after restricting participants to those who were diagnosed with CRC after 11 years of recruitment, positive associations of dietary AGEs with increased mortality risk were observed. These results are consistent with our original hypotheses. A possibility for this could be that AGEs might influence molecular features and specific molecular pathways of colon cancer during its development and progression [49], so that earlier AGE exposures may be more important than later ones in relation to mortality risk. We also found a suggestive positive association between intakes of CML and CEL and high mortality risk with more than two years of follow-up after cancer diagnosis, which suggests that the exposure might be more relevant for long-term outcomes. We also found a positive association between intake of AGEs and high mortality risk in participants without type II diabetes, which could in part be due to higher formation of endogenous AGEs relative to dietary intake of AGEs among individuals with type II diabetes [9]. 

The major strengths of this study include the prospective study design, large sample size, comprehensive collection and assessment of multiple potential confounding/effect modifying factors, and multiple sensitivity analyses. In addition, our study used a state-of-the-art quantitative instrumental mass-spectrometry-based method to assess three different types of AGEs in foods [35], and we followed the recent “quality control” recommendations for studies on AGEs, i.e., the study of several specific AGEs and the use of a validated food composition database to estimate individual dietary AGE exposures [50].

Our study also has several limitations. First, the dietary data were measured at baseline before cancer diagnosis, and some participants might have modified their diet after CRC diagnosis. However, earlier (not later) exposure to AGEs could be more important in shaping the microenvironment for tumor development and determining its aggressiveness. Second, we cannot exclude a potential misclassification in estimating dietary AGEs, which is also influenced by personal cooking preferences. However, it was reported previously that higher intakes of CEL, CML, and MG-H1 were positively associated with weight gain and obesity after, on average, 5 years of follow-up in the EPIC study, which indicates the validity of our dietary AGE assessment [36]. Third, our study population was limited to Western European CRC patients, which restricts the generalizability of the study results. Fourth, CRC cases might have changed their diet after cancer diagnosis. However, the pre-diagnostic diet could be more indicative of exposures that promoted tumor development and progression and influenced the tumor molecular profile and aggressiveness. Fifth, we did not have information on CRC treatment. Generally, during the follow-up period, we would not expect CRC treatment to differ by country within the countries and centers participating in this study or by year of diagnosis or tumor stage. Therefore, our analyses were conducted stratified by country of CRC diagnosis and adjusted for tumor stage and year of diagnosis. Finally, to estimate the effect of missing CRC stage data we used several approaches, all of which demonstrated the robustness of effect estimates against uncertainties in CRC stage classification.

## 5. Conclusions

In conclusion, our findings suggest that pre-diagnostic dietary intakes of AGEs might not be associated with CRC-specific or all-cause mortality among individuals diagnosed with CRC. Further studies are necessary to investigate these associations in different populations and examine whether these associations are stronger among women and long-term survivors and differ by diabetes status and timing of exposure to AGEs. 

## Figures and Tables

**Table 1:** Selected baseline characteristics of CRC cases (*N* = 5801) according to quintiles of pre-diagnostic combined ^a^ energy-adjusted dietary AGE intake ^b^ in the EPIC study.

Characteristic	Combined ^a^ Dietary AGEs (mg/d)					
	Quintile 1: <19.79 (*N* = 1160)	Quintile 2: 19.79–23.20 (*N* = 1160)	Quintile 3: 23.21–26.80 (*N* = 1161)	Quintile 4: 26.81–32.26 (*N* = 1160)	Quintile 5: >32.26 (*N* = 1160)	*p*-Value
Age at diagnosis, mean (SD), y	66.04 (7.68)	65.69 (8.63)	66.01 (8.65)	66.49 (8.70)	67.25 (8.93)	*0.39*
Women, *N* (%)	870 (75)	709 (61)	680 (59)	570 (49)	491 (42)	*<0.001*
Year at dx, median (min-max)	2004 (1993–2013)	2005 (1993–2013)	2005 (1993–2013)	2005 (1993–2013)	2005 (1992–2013)	*0.89*
Stage of disease, *N* (%) ^c^						*<0.001*
I	225 (19)	217 (19)	228 (20)	259 (22)	226 (19)	
II	241 (21)	224 (19)	177 (15)	213 (18)	172 (15)	
III	286 (25)	304 (26)	325 (28)	258 (22)	250 (22)	
IV	165 (14)	142 (12)	128 (11)	116 (10)	98 (8)	
Location of primary tumor, *N* (%)						*0.75*
Colon	762 (66)	748 (65)	766 (66)	750 (65)	752 (65)	
Rectum	398 (34)	412 (35)	395 (34)	410 (35)	408 (35)	
Smoking status, *N* (%) ^c^						*<0.001*
Never	462 (40)	482 (42)	462 (40)	471 (41)	494 (43)	
Former	364 (31)	365 (31)	403 (35)	410 (35)	422 (36)	
Current	326 (28)	301 (26)	276 (24)	259 (22)	224 (19)	
BMI, mean (SD), kg/m^2^	26.14 (4.33)	26.29 (4.21)	26.34 (4.16)	26.54 (4.16)	26.17 (4.26)	*0.15*
Physical activity ^d^, *N* (%) ^c^						*<0.001*
Inactive	197 (17)	182 (16)	187 (16)	181 (16)	128 (11)	
Moderately inactive	383 (33)	375 (32)	351 (30)	320 (28)	347 (30)	
Moderately active	446 (38)	450 (39)	458 (40)	458 (39)	438 (38)	
Active	85 (7)	105 (9)	99 (9)	103 (9)	123 (11)	
Diabetes ^e^, *N* (%) ^c^						*<0.001*
No	952 (82)	943 (81)	922 (80)	901 (78)	881 (76)	
Yes	105 (9)	106 (9)	122 (11)	111 (10)	105 (9)	
Daily dietary intakes						
Total energy, mean (SD), kcal	2019.4 (584.6)	2098.2 (609.6)	2115.0 (602.4)	2158.6 (628.8)	2155.3 (639.2)	*<0.001*
Fiber, mean (SD), g	19.8 (6.9)	21.6 (6.8)	22.8 (7.1)	23.9 (7.7)	25.4 (8.7)	*<0.001*
Dietary calcium, mean (SD), mg	980.4 (440.7)	976.7 (408.5)	969.8 (378.6)	989.4 (375.1)	1003.4 (394.1)	*0.30*
Fruits, mean (SD), g	220.6 (191.4)	223.3 (184.7)	225.2 (183.0)	222.1 (161.4)	202.9 (155.0)	*0.02*
Vegetables, mean (SD), g	179.9 (123.3)	178.7 (115.9)	187.0 (115.0)	193.7 (125.2)	191.7 (132.1)	*0.007*
Red meat, mean (SD), g	50.9 (35.3)	52.7 (38.0)	52.7 (41.1)	46.5 (37.6)	41.9 (37.4)	*<0.001*
Processed meat, mean (SD), g	30.9 (26.7)	34.5 (28.8)	34.6 (31.3)	37.8 (30.5)	37.2 (36.1)	*<0.001*

Abbreviations: AGE, advanced glycation end product; BMI, body mass index; CRC, colorectal cancer; CML, Ne-(caroxymethyl)lysine; CEL, Ne-(1-caroxyethyl)lysine; d, day; g, gram; MG-H1, Ne-(5-hydro-5-methyl-4-imidazolon-2-yl)-ornithine; SD, standard deviation; y, years; dx, diagnosis. ^a^ Combined AGEs = CML+CEL+MG-H1. ^b^ Total energy-adjusted residuals were computed by fitting a linear regression of the log-transformed intake of AGEs on total energy intake, sex, and center. ^c^ The sum of percentages across subgroups did not add up to 100% due to missing values. ^d^ Combined recreational and household activity as measured by the Cambridge index and shown as sex-specific categories of metabolic equivalents. ^e^ Diabetes based on self-reporting at baseline and ascertainment during follow-up.

**Table 2:** The associations of pre-diagnostic energy-adjusted dietary intakes of advanced glycation end products (AGEs) ^a^ with all-cause and CRC-specific mortality among CRC patients in the EPIC study (*n* = 5801).

AGEs ^a^	Cut-Offs	*N*	All-Cause Mortality			CRC-Specific Mortality		
			Event	HR (95% CI) ^b,c^	HR (95% CI) ^b,d^	Event	HR (95% CI) ^b,c^	HR (95% CI) ^b,d^
CML, mg/d								
Quintile 1	<2.3	1160	447	1.00 (ref)	1.00 (ref)	348	1.00 (ref)	1.00 (ref)
Quintile 2	[2.3–2.7)	1160	489	1.11 (0.97–1.26)	1.14 (1.00–1.30)	384	1.13 (0.98–1.31)	1.13 (0.98–1.32)
Quintile 3	[2.7–3.1)	1161	498	1.11 (0.97–1.27)	1.13 (0.99–1.30)	368	1.08 (0.93–1.26)	1.10 (0.94–1.28)
Quintile 4	[3.1–3.7)	1160	474	0.99 (0.86–1.14)	1.02 (0.89–1.18)	361	1.03 (0.88–1.21)	1.04 (0.89–1.22)
Quintile 5	≥3.7	1160	513	1.08 (0.93–1.25)	1.13 (0.98–1.30)	380	1.14 (0.97–1.34)	1.16 (0.98–1.36)
*p_trend_* ^e^				*0.72*	*0.36*		*0.31*	*0.23*
Per 1.01 mg/d				1.00 (0.96, 1.04)	1.02 (0.98–1.06)		1.03 (0.98–1.08)	1.03 (0.98, 1.08)
CEL, mg/d								
Quintile 1	<1.6	1160	463	1.00 (ref)	1.00 (ref)	354	1.00 (ref)	1.00 (ref)
Quintile 2	[1.6–1.9)	1160	435	0.90 (0.79–1.03)	0.92 (0.80–1.05)	335	0.92 (0.79–1.07)	0.93 (0.80–1.09)
Quintile 3	[1.9–2.2)	1161	492	1.01 (0.88–1.15)	1.01 (0.89–1.15)	378	1.04 (0.89–1.21)	1.04 (0.89–1.21)
Quintile 4	[2.2–2.6)	1160	496	0.92 (0.80–1.06)	0.92 (0.80–1.06)	368	0.96 (0.82–1.13)	0.96 (0.82–1.13)
Quintile 5	≥2.6	1160	535	1.03 (0.89–1.18)	1.03 (0.89–1.19)	406	1.10 (0.93–1.29)	1.11 (0.94–1.31)
*p_trend_* ^e^				*0.45*	*0.47*		*0.14*	*0.13*
Per 0.73 mg/d				1.01 (0.97–1.06)	1.01 (0.97–1.06)		1.02 (0.97–1.08)	1.02 (0.97–1.08)
MG-H1, mg/d								
Quintile 1	<15.5	1160	457	1.00 (ref)	1.00 (ref)	360	1.00 (ref)	1.00 (ref)
Quintile 2	[15.5–18.4)	1160	471	1.07 (0.93–1.22)	1.07 (0.94–1.22)	370	1.07 (0.93–1.25)	1.08 (0.93–1.26)
Quintile 3	[18.4–21.4)	1161	460	0.99 (0.87–1.14)	1.00 (0.88–1.15)	335	0.94 (0.80–1.09)	0.95 (0.81–1.11)
Quintile 4	[21.4–26.1)	1160	506	1.08 (0.94–1.23)	1.09 (0.95–1.24)	393	1.07 (0.92–1.25)	1.09 (0.93–1.27)
Quintile 5	≥26.1	1160	527	1.07 (0.93–1.22)	1.09 (0.95–1.25)	383	1.08 (0.92–1.26)	1.10 (0.94–1.28)
*p_trend_* ^e^				*0.37*	*0.23*		*0.35*	*0.24*
Per 8.47 mg/d				1.01 (0.97–1.05)	1.02 (0.98–1.05)		1.02 (0.98–1.07)	1.03 (0.98–1.08)
Combined AGEs ^f^, mg/d								
Quintile 1	<19.8	1160	453	1.00 (ref)	1.00 (ref)	356	1.00 (ref)	1.00 (ref)
Quintile 2	[19.8–23.2)	1160	467	1.08 (0.94–1.23)	1.09 (0.95–1.24)	367	1.08 (0.93–1.26)	1.10 (0.95–1.28)
Quintile 3	[23.2–26.8)	1161	463	1.01 (0.88–1.15)	1.02 (0.89–1.17)	336	0.96 (0.82–1.12)	0.97 (0.83–1.13)
Quintile 4	[26.8–32.3)	1160	520	1.09 (0.95–1.24)	1.10 (0.96–1.26)	405	1.10 (0.95–1.28)	1.11 (0.96–1.30)
Quintile 5	≥32.3	1160	518	1.06 (0.92–1.21)	1.08 (0.94–1.24)	377	1.07 (0.91–1.25)	1.09 (0.93–1.28)
*p_trend_* ^e^				*0.50*	*0.33*		*0.42*	*0.29*
Per 9.83 mg/d				1.01 (0.97–1.05)	1.02 (0.98–1.05)		1.02 (0.98–1.08)	1.03 (0.98–1.08)

Abbreviations: AGE, advanced glycation end products; CRC, colorectal cancer; CML, Ne-(caroxymethyl)lysine; CEL, Ne-(1-caroxyethyl)lysine; d, day; MGH1, Ne-(5-hydro-5-methyl-4-imidazolon-2-yl)-ornithine; mg, milligram; HR, hazard ratio; ref, reference category. ^a^ Residuals were computed by a linear regression of the log-transformed intake of AGEs on total energy intake, sex and center. ^b^ Quintile 1 was a reference category in each model. ^c^ Multivariable cox proportional hazard model, stratified by center, and adjusted for sex, age at diagnosis (yrs; continuous) and stage (categorical) and total energy intake (kcal/d; continuous). ^d^ Multivariable cox proportional hazard model, stratified by center, and adjusted for sex, age at diagnosis (y; continuous), stage (categorical), total energy intake (kcal/d; continuous), year of diagnosis (continuous), location of tumor(categorical), BMI (continuous), smoking status(categorical) and prevalent/incident diabetes (categorical). ^e^ P_trend_ was calculated with the median value of each quintile of AGE as a continuous variable, adjusted for covariates in the corresponding model. ^f^ Combined AGEs: CML+CEL+MGH1.

## Data Availability

Data supporting reported results are available from the corresponding author upon request.

## Supplementary Materials

The following are available online at https://www.mdpi.com/article/10.3390/nu13124435/s1, Table S1: The associations of pre-diagnostic dietary energy-adjusted intakes of advanced glycation end products (AGEs) with all-cause and CRC mortality by sex and among men and women with CRC combined for sex-specific quintiles and per one SD change, the EPIC study (*n* = 5801), Table S2: Adjusted HRs and 95% CIs for an increment of per one SD change of dietary advanced glycation end products (AGEs) and CRC-specific mortality across strata of potential effect modifiers among CRC patients in the EPIC study (*n* = 5801), Table S3: Adjusted HRs and 95% CIs for an increment of per one SD change of dietary advanced glycation end products (AGEs) and all-cause mortality across strata of potential effect modifiers among CRC patients in the EPIC study (*n* = 5801), Figure S1: Non-parametrical restricted cubic splines of AGEs with CRC-specific mortality among CRC patients in the EPIC study, Figure S2: Non-parametrical restricted cubic splines of AGEs with all-cause mortality among CRC patients in the EPIC study.

## Disclaimer

Where authors are identified as personnel of the International Agency for Research on Cancer/World Health Organization, the authors alone are responsible for the views expressed in this article and they do not necessarily represent the decisions, policy, or views of the International Agency for Research on Cancer/World Health Organization. The funding sources had no influence on the design of the study; the collection, analysis, and interpretation of data; the writing of the report; or the decision to submit the paper for publication.
