# Supplementary material for: Dietary Intake of Advanced Glycation End Products (AGEs) and Mortality among Individuals with Colorectal Cancer
Source: Nutrients. 2021 Dec 10;13(12):4435. doi: 10.3390/nu13124435 (PMC8704988; doi:10.3390/nu13124435)
Supplement: Supplementary file 1 [file nutrients-13-04435-s001.zip › nutrients-1490031-supplementary.pdf]

nutrients

MDPI

Supplemental Materials

Dietary Intake of Advanced Glycation End Products (AGEs) and Mortality Among Individuals With Colorectal Cancer

Table S1. The associations of pre-diagnostic dietary energy-adjusted intakes of advanced glycation end products (AGEs)[a] with all-cause and CRC mortality by sex and among men and women with CRC combined for sex-specific quintiles and per one SD change, the EPIC study (n = 5,801).

Sex-Specific Quintiles	Men				Women				$P_{interaction}$[c,d]	Combined		
	Cut-offs	*N*	Events	HR (95%CI)[b,c]	Cut-offs	*N*	Events	HR (95%CI)[b,c]		*N*	Events	HR (95%CI)[b,c]
CML, mg/d												
All-cause mortality									*0.13*			
1	<2.6	496	234	1.00 (ref)	<2.1	664	247	1.00 (ref)		1,160	481	1.00 (ref)
2	[2.6-3.0)	496	240	1.11 (0.93-1.34)	[2.1-2.5)	664	254	1.03 (0.86-1.23)		1,160	494	1.05 (0.92-1.19)
3	[3.0-3.5)	497	219	0.93 (0.77-1.12)	[2.5-2.9)	664	264	1.13 (0.94-1.35)		1,161	483	1.02 (0.89-1.16)
4	[3.5-4.1)	496	207	0.95 (0.78-1.16)	[2.9-3.4)	664	261	1.11 (0.92-1.34)		1,160	468	1.02 (0.89-1.17)
5	≥4.1	496	232	0.97 (0.80-1.18)	≥3.4	664	263	1.06 (0.88-1.29)		1,160	495	1.02 (0.89-1.16)
p_{trend}												
Per 1.01 mg/d				0.99 (0.94-1.04)				1.06 (0.99-1.13)	*0.10*			1.02 (0.98-1.06)
CRC-specific mortality									*0.56*			
1	<2.6	496	166	1.00 (ref)	<2.1	664	196	1.00 (ref)		1,160	362	1.00 (ref)
2	[2.6-3.0)	496	181	1.15 (0.93-1.43)	[2.1-2.5)	664	212	1.04 (0.85-1.27)		1,160	393	1.07 (0.93-1.24)
3	[3.0-3.5)	497	151	0.95 (0.76-1.18)	[2.5-2.9)	664	202	1.06 (0.86-1.30)		1,161	353	1.00 (0.86-1.16)
4	[3.5-4.1)	496	165	1.05 (0.84-1.31)	[2.9-3.4)	664	203	1.08 (0.87-1.33)		1,160	368	1.06 (0.91-1.23)
5	≥4.1	496	168	1.07 (0.85-1.34)	≥3.4	664	197	1.00 (0.80-1.25)		1,160	365	1.04 (0.89-1.21)
p_{trend}												
Per 1.01 mg/d				1.02 (0.95-1.09)				1.04 (0.96-1.13)	*0.55*			1.03 (0.98-1.08)
CEL, mg/d												
All-cause mortality									*0.59*			
1	<1.9	496	220	1.00 (ref)	<1.5	664	252	1.00 (ref)		1,160	472	1.00 (ref)
2	[1.9-2.1)	496	229	1.03 (0.85-1.25)	[1.5-1.8)	664	241	0.94 (0.79-1.13)		1,160	470	0.99 (0.87-1.12)
3	[2.1-2.4)	497	216	0.89 (0.73-1.08)	[1.8-2.0)	664	253	1.02 (0.85-1.22)		1,161	469	0.95 (0.83-1.08)
4	[2.4-2.9)	496	226	1.00 (0.82-1.22)	[2.0-2.4)	664	264	1.03 (0.86-1.24)		1,160	490	1.01 (0.89-1.16)

5	≥2.9	496	241	1.01 (0.83-1.24)	≥2.4	664	279	1.01 (0.83-1.22)		1,160	520	1.01 (0.88-1.16)
p_{trend}												
Per 0.73 mg/d				0.99 (0.94-1.05)				1.05 (0.97-1.12)	*0.40*			1.01 (0.97-1.06)
CRC-specific mortality									*0.97*			
1	<1.9	496	154	1.00 (ref)	<1.5	664	201	1.00 (ref)		1,160	355	1.00 (ref)
2	[1.9-2.1)	496	165	1.02 (0.82-1.28)	[1.5-1.8)	664	197	0.96 (0.78-1.17)		1,160	362	0.99 (0.86-1.16)
3	[2.1-2.4)	497	161	0.97 (0.77-1.22)	[1.8-2.0)	664	191	0.96 (0.78-1.19)		1,161	352	0.96 (0.83-1.12)
4	[2.4-2.9)	496	177	1.12 (0.89-1.41)	[2.0-2.4)	664	212	1.07 (0.87-1.31)		1,160	389	1.09 (0.94-1.27)
5	≥2.9	496	174	1.07 (0.84-1.37)	≥2.4	664	209	0.99 (0.80-1.23)		1,160	383	1.03 (0.88-1.21)
p_{trend}												
Per 0.73 mg/d				1.01 (0.95-1.08)				1.04 (0.96-1.13)	*0.67*			1.02 (0.97-1.08)
MG-H1, mg/d												
All-cause mortality									*0.37*			
1	<16.6	496	154	1.00 (ref)	<14.8	664	251	1.00 (ref)		1,160	405	1.00 (ref)
2	[16.6-19.6)	496	175	1.08 (0.90-1.31)	[14.8-17.5)	664	233	0.96 (0.80-1.15)		1,160	408	1.00 (0.88-1.14)
3	[19.6-23.0)	497	172	0.96 (0.79-1.16)	[17.5-20.3)	664	244	1.05 (0.88-1.27)		1,161	416	1.00 (0.87-1.14)
4	[23.0-27.9)	496	162	1.02 (0.84-1.24)	[20.3-24.7)	664	280	1.13 (0.94-1.36)		1,160	442	1.07 (0.93-1.22)
5	≥27.9	496	168	0.92 (0.75-1.11)	≥24.7	664	281	1.19 (0.99-1.44)		1,160	449	1.04 (0.91-1.19)
p_{trend}												
Per 8.47 mg/d									*0.11*			
CRC-specific mortality									*0.57*			
1	<16.6	496	154	1.00 (ref)	<14.8	664	208	1.00 (ref)		1,160	362	1.00 (ref)
2	[16.6-19.6)	496	175	1.07 (0.88-1.29)	[14.8-17.5)	664	185	0.91 (0.74-1.12)		1,160	360	0.98 (0.84-1.13)
3	[19.6-23.0)	497	172	0.95 (0.79-1.16)	[17.5-20.3)	664	196	1.02 (0.83-1.25)		1,161	368	1.00 (0.86-1.17)
4	[23.0-27.9)	496	162	1.00 (0.82-1.21)	[20.3-24.7)	664	210	1.03 (0.84-1.27)		1,160	372	1.01 (0.87-1.18)
5	≥27.9	496	168	0.89 (0.73-1.08)	≥24.7	664	211	1.14 (0.92-1.40)		1,160	379	1.05 (0.90-1.22)
p_{trend}												
Per 8.47 mg/d				0.98 (0.93-1.03)				1.07 (1.00-1.15)	*0.35*			1.03 (0.98-1.08)

					Combined AGEs[e], mg/d							
All-cause mortality									*0.31*			
1	<21.6	496	213	1.00 (ref)	<18.7	664	253	1.00 (ref)		1,160	466	1.00 (ref)
2	[21.6-24.9)	496	239	1.15 (0.95-1.39)	[18.7-21.9)	664	243	0.97 (0.81-1.17)		1,160	482	1.04 (0.91-1.19)
3	[24.9-28.9)	497	226	1.02 (0.84-1.24)	[21.9-25.2)	664	235	0.98 (0.82-1.19)		1,161	461	0.99 (0.87-1.13)
4	[28.9-34.6)	496	222	0.99 (0.81-1.20)	[25.2-29.9)	664	275	1.09 (0.91-1.31)		1,160	497	1.04 (0.91-1.19)
5	≥34.6	496	232	0.98 (0.80-1.19)	≥29.9	664	283	1.17 (0.97-1.41)		1,160	515	1.07 (0.93-1.22)
p_{trend}												
Per 9.83 mg/d				0.98 (0.94-1.03)				1.08 (1.01-1.16)	*0.11*			1.02 (0.98-1.05)
CRC-specific mortality									*0.14*			
1	<21.6	496	147	1.00 (ref)	<18.7	664	210	1.00 (ref)		1,160	357	1.00 (ref)
2	[21.6-24.9)	496	186	1.28 (1.03-1.60)	[18.7-21.9)	664	194	0.95 (0.78-1.16)		1,160	380	1.08 (0.93-1.26)
3	[24.9-28.9)	497	172	1.15 (0.92-1.44)	[21.9-25.2)	664	183	0.93 (0.75-1.14)		1,161	355	1.02 (0.87-1.18)
4	[28.9-34.6)	496	159	1.03 (0.82-1.30)	[25.2-29.9)	664	213	1.03 (0.84-1.26)		1,160	372	1.03 (0.88-1.2P)
5	≥34.6	496	167	1.09 (0.86-1.38)	≥29.9	664	210	1.12 (0.91-1.38)		1,160	377	1.09 (0.94-1.28)
p_{trend}												
Per 9.83 mg/d				1.00 (0.93-1.07)				1.07 (0.99-1.15)	*0.36*			1.03 (0.98-1.08)

Abbreviation: CRC, colorectal cancer; CML, Ne-(caroxymethyl)lysine ; CEL, Ne-(1-caroxyethyl)lysine; MG-H1, Ne-(5-hydro-5-methyl-4-imidazolon-2-yl)-ornithine; mg, milligram; HR, hazard ratio; ref., reference category. [a] Residuals were computed by a linear regression of the log-transformed intake of AGEs on total energy intake, sex and center. [b] Quintile 1 was a reference category in each model. [c] Multivariable cox proportional hazard model, stratified by center, and adjusted for sex, age at diagnosis (yrs; continuous), stage (categorical), total energy intake (kcal/d; continuous), year of diagnosis (continuous), location of tumor(categorical), BMI (continuous), smoking status(categorical) and prevalent/incident diabetes (categorical). [d] P_{trend} was calculated with the median value of each quintile of AGE as a continuous variable, adjusted for covariates in the corresponding model. [e] Combined AGEs: CML+CEL+MG-H1.

Table S2. Adjusted HRs and 95% CIs for an increment of per one SD[a] change of dietary advanced glycation end products (AGEs)[b] and CRC-specific mortality across strata of potential effect modifiers among CRC patients in the EPIC study (n = 5,801).

Sensitivity Analysis/Potential Effect Modifier	Events/Total	CML		CEL		MG-H1		Combined AGEs[c]	
		HR (95% CI)[d]	$P_{interaction}$ or P_{trend}	HR (95% CI)[d]	$P_{interaction}$ or P_{trend}	HR (95% CI)[d]	$P_{interaction}$ or P_{trend}	HR (95% CI)[d]	$P_{interaction}$ or P_{trend}
All participants	1,841/5801	1.03(0.98-1.08)	*0.25*[e]	1.02(0.97-1.08)	*0.35*[e]	1.03(0.98-1.08)	*0.25*[e]	1.03(0.98-1.08)	*0.24*[e]
Complete CRC stage data	1,329/4,254	0.98(0.91-1.05)	*0.54*[e]	1.00(0.93-1.07)	*0.94*[e]	0.97(0.91-1.04)	*0.39*[e]	0.98(0.91-1.04)	*0.42*[e]
Imputed CRC stage data[f]	1,841/5,801	1.02(0.96-1.07)	*0.55*[e]	1.02(0.96-1.07)	*0.42*[e]	1.02(0.96-1.07)	*0.50*[e]	1.02(0.96-1.07)	*0.49*[e]
Time between recruitment and CRC dx, yrs									
< 6.4	758/1,931	0.93(0.85-1.02)	*0.008*	0.94(0.86-1.02)	*0.01*	0.93(0.85-1.01)	*0.01*	0.93(0.85-1.01)	*0.003*
6.4-11.2	618/1,940	1.04(0.96-1.13)		1.06(0.96-1.16)		1.04(0.96-1.13)		1.04(0.96-1.13)	
≥ 11.2	465/1,930	1.09(1.00-1.20)		1.11(1.02-1.20)		1.11(1.02-1.21)		1.11(1.03-1.21)	
Follow-up, yrs[g]									
≥ 2	772/4,170	1.09(1.01-1.17)	*0.03*[e]	1.07(1.00-1.15)	*0.07*[e]	1.06(0.98-1.14)	*0.14*[e]	1.06(0.99-1.14)	*0.11*[e]
≥ 3	500/3,590	1.10(1.00-1.20)	*0.05*[e]	1.07(0.98-1.17)	*0.15*[e]	1.04(0.95-1.14)	*0.42*[e]	1.05(0.96-1.15)	*0.32*[e]
≥ 5	202/2,684	1.19(1.04-1.36)	*0.01*[e]	1.15(1.02-1.30)	*0.02*[e]	1.09(0.95-1.25)	*0.22*[e]	1.11(0.97-1.26)	*0.14*[e]
Sex									
Women	1,010/3,320	1.04(0.96-1.13)	*0.55*	1.04(0.96-1.13)	*0.67*	1.07(1.00-1.15)	*0.35*	1.07(0.99-1.15)	*0.36*
Men	831/2,481	1.02(0.95-1.09)		1.01(0.95-1.08)		0.99(0.93-1.06)		1.00(0.93-1.07)	
Age at dx, yrs									
< 66.4	921/2,897	1.02(0.94-1.10)	*0.85*	1.05(0.97-1.13)	*0.96*	1.03(0.95-1.11)	*0.56*	1.03(0.96-1.11)	*0.57*
≥ 66.4	920/2,904	1.04(0.94-1.11)		1.01(0.94-1.08)		1.03(0.97-1.10)		1.03(0.97-1.10)	
Anatomical site									
Colon	1,184/3,778	1.07(1.01-1.13)	*0.52*	1.05(0.99-1.12)	*0.91*	1.05(0.99-1.11)	*0.52*	1.05(0.99-1.12)	*0.55*
Rectum	657/2,023	0.99(0.91-1.07)		1.01(0.93-1.10)		1.02(0.94-1.11)		1.02(0.94-1.10)	
Colon subsite[h]									
Proximal	572/1,827	1.08(0.99-1.17)	*0.95*	1.03(0.94-1.13)	*0.52*	1.07(0.99-1.16)	*0.72*	1.07(0.99-1.16)	*0.79*
Distal	492/1,697	1.10(0.99-1.21)		1.07(0.97-1.18)		1.05(0.95-1.15)		1.05(0.96-1.16)	
Stage[i]									
I and II	332/2,182	0.96(0.84-1.09)	*0.18*	1.02(0.90-1.15)	*0.10*	0.96(0.85-1.09)	*0.22*	0.97(0.85-1.10)	*0.17*
III and IV	997/2,072	0.93(0.86-1.02)		0.93(0.86-1.04)		0.93(0.85-1.01)		0.93(0.85-1.01)	
BMI, kg/m^2									
< 25	739/2,437	1.06(0.98-1.14)	*0.92*	1.03(0.94-1.12)	*0.69*	1.02(0.94-1.10)	*0.43*	1.02(0.94-1.10)	*0.46*
25-29.9	776/2,406	1.00(0.93-1.09)		1.03(0.95-1.11)		1.03(0.96-1.11)		1.03(0.96-1.11)	
≥ 30	325/951	1.06(0.94-1.20)		1.01(0.90-1.13)		1.11(0.99-1.25)		1.10(0.98-1.24)	
Physical activity									
Inactive	258/875	1.12(0.96-1.31)	*0.08*	1.10(0.94-1.27)	*0.25*	1.13(0.96-1.32)	*0.13*	1.13(0.97-1.32)	*0.13*
Moderately inactive	554/1,776	0.97(0.88-1.06)		0.97(0.88-1.07)		1.00(0.91-1.10)		0.99(0.91-1.09)	
Moderately active	753/2,250	1.01(0.93-1.09)		0.98(0.90-1.07)		1.02(0.94-1.10)		1.01(0.94-1.09)	
Active	156/515	1.06(0.87-1.30)		1.18(0.93-1.48)		0.96(0.78-1.19)		0.98(0.80-1.21)	
Smoking status									
Never	741/2,371	1.06(0.97-1.14)	*0.83*	1.04(0.96-1.13)	*0.21*	1.06(0.99-1.15)	*0.60*	1.06(0.99-1.15)	*0.75*
Former	603/1,964	1.01(0.93-1.10)		1.01(0.92-1.10)		1.02(0.94-1.10)		1.01(0.94-1.09)	
Current	466/1,386	1.00(0.89-1.13)		0.99(0.88-1.11)		0.97(0.86-1.10)		0.97(0.86-1.10)	
Alcohol intake									
Non-drinkers	142/373	1.10(0.95-1.27)	*0.62*	1.07(0.90-1.29)	*0.53*	1.07(0.93-1.24)	*0.62*	1.08(0.93-1.24)	*0.63*
< sex-specific median[j]	833/2,527	1.01(0.94-1.08)		1.01(0.94-1.09)		1.01(0.95-1.08)		1.01(0.95-1.08)	
≥ sex-specific median[j]	866/2,901	1.00(0.92-1.09)		1.00(0.92-1.08)		1.01(0.92-1.10)		1.01(0.92-1.10)	

Diabetes[k]									
Yes	165/549	0.89(0.94-1.06)	*0.02*	0.93(0.78-1.11)	*0.03*	0.90(0.75-1.07)	*0.08*	0.90(0.75-1.07)	*0.06*
No	1,432/4,599	1.07(1.01-1.13)		1.05(1.00-1.12)		1.06(1.00-1.12)		1.06(1.00-1.12)	

Abbreviation: AGE, advanced glycation endproducts; BMI, body mass index; CML, Ne-(caroxymethyl)lysine ; CEL, Ne-(1-caroxyethyl)lysine; dx, diagnosis; CRC, colorectal cancer; MG-H1, Ne-(5-hydro-5-methyl-4-imidazolon-2-yl)-ornithine; SD, standard deviation; yrs, years. [a] SD for CML = 1.01 mg/d; SD for CEL = 0.73 mg/d; SD for MG-H1 = 8.47 mg/d; and SD for the combined AGEs = 9.83 mg/d. [b] Residuals were computed by a linear regression of the log-transformed intake of AGEs on total energy intake, sex and center. [c] Combined AGEs: CML+CEL+MG-H1. [d] Multivariable cox proportional hazard model, adjusted for sex, age at diagnosis (yrs; continuous), stage (categorical), total energy intake (kcal/d; continuous), year of diagnosis (continuous), location of tumor(categorical), BMI (continuous), smoking status (categorical) and prevalent/incident diabetes (categorical), and stratified by center. In the stratified models, the stratifying variable was not adjusted for. P for interaction is provided unless otherwise indicated. [e] P_{trend}. [f] Missing stage data were imputed using the algorithm described in the Statistics analyses section.[g] Multivariable model including all CRC cases with time interval between age at dx and age at end of follow up, of more than 2,3, and 5 years. [h] Only colon tumors with known locations included. Unspecified and overlapping (n = 254) were excluded. [i] Missing stage variables were not included. [j] Median of alcohol intake for men: 15.7 g/d, for women: 3.7 g/d. [k] Diabetes based on self-report at baseline and ascertainment during follow-up.

Table S3. Adjusted HRs and 95% CIs for an increment of per one SD[a] change of dietary advanced glycation end products (AGEs)[b] and all-cause mortality across strata of potential effect modifiers among CRC patients in the EPIC study (n = 5,801).

Sensitivity Analysis/ Potential Effect Modifier	Events /Total	CML		CEL		MGH1		Combined AGEs[c]	
		HR (95% CI)[d]	$p_{interaction}$ or p_{trend}	HR (95% CI)[d]	$p_{interaction}$ or p_{trend}	HR (95% CI)[d]	$p_{interaction}$ or p_{trend}	HR (95% CI)[d]	$p_{interaction}$ or p_{trend}
All participants	2,421/5,801	1.02(0.98-1.06)	*0.38*[e]	1.01(0.97-1.06)	*0.56*[e]	1.02(0.98-1.05)	*0.41*[e]	1.02(0.98-1.05)	*0.40*[e]
Sensitivity analysis									
Complete CRC stage data	1,711/4,254	0.98(0.93-1.04)	*0.52*[e]	1.00(0.94-1.05)	*0.88*[e]	0.98(0.93-1.05)	*0.33*[e]	0.98(0.93-1.03)	*0.35*[e]
Imputed CRC stage data[f]	2,421/5,801	1.02(0.96-1.07)	*0.55*[e]	1.02(0.96-1.07)	*0.42*[e]	1.02(0.96-1.07)	*0.50*[e]	1.02(0.96-1.07)	*0.49*[e]
Time between recruitment and CRC dx, yrs									
< 6.4	991/1,931	0.97(0.91-1.03)	*0.02*	0.98(0.91-1.05)	*0.05*	0.97(0.91-1.03)	*0.01*	0.97(0.91-1.03)	*0.01*
6.4-11.2	813/1,940	1.06(0.98-1.14)		1.03(0.95-1.12)		1.04(0.97-1.12)		1.04(0.97-1.12)	
≥ 11.2	617/1,930	1.05(0.97-1.14)		1.06(0.98-1.14)		1.07(0.99-1.15)		1.07(0.99-1.15)	
Follow-up[g], yrs									
≥ 2	1,158/4,170	1.05(0.99-1.10)	*0.09*[e]	1.05(0.99-1.11)	*0.14*[e]	1.03(0.98-1.08)	*0.27*[e]	1.03(0.98-1.08)	*0.22*[e]
≥ 3	831/3,590	1.06(1.00-1.12)	*0.05*[e]	1.04(0.98-1.12)	*0.21*[e]	1.02(0.97-1.08)	*0.43*[e]	1.03(0.97-1.09)	*0.34*[e]
≥ 5	439/2,684	1.07(1.00-1.15)	*0.04*[e]	1.09(1.01-1.19)	*0.06*[e]	1.03(0.97-1.10)	*0.37*[e]	1.04(0.97-1.10)	*0.28*[e]
Sex			*0.10*		*0.40*		*0.11*		*0.11*
Women	1,289/3,320	1.06(1.00-1.13)		1.05(0.97-1.12)		1.08(1.01-1.15)		1.08(1.01-1.15)	
Men	1,132/2,481	0.99(0.94-1.05)		0.99(0.94-1.05)		0.98(0.94-1.03)		0.98(0.94-1.03)	
Age at dx, yrs									
< 66.4	1,112/2,897	1.03(0.96-1.10)	*0.52*	1.06(0.99-1.13)	*0.77*	1.04(0.97-1.12)	*0.76*	1.04(0.97-1.12)	*0.76*
≥ 66.4	1,309/2,904	1.01(0.96-1.06)		0.98(0.93-1.04)		1.01(0.97-1.06)		1.01(0.96-1.06)	
Anatomical site									
Colon	1,584/3,778	1.04(0.99-1.08)	*0.95*	1.04(0.99-1.09)	*0.94*	1.02(0.98-1.07)	*0.88*	1.03(0.96-1.11)	*0.90*
Rectum	837/2,023	1.01(0.94-1.09)		1.02(0.95-1.10)		1.03(0.96-1.11)		1.03(0.96-1.11)	
Colon subsite[h]									
Proximal	788/1,827	1.09(1.01-1.16)	*0.24*	1.06(0.99-1.14)	*0.70*	1.08(1.01-1.15)	*0.18*	1.08(1.01-1.15)	*0.19*
Distal	641/1,697	1.04(0.97-1.11)		1.04(0.96-1.12)		1.01(0.95-1.08)		1.01(0.95-1.08)	
Stage[i]									
I and II	507/2,182	0.93(0.83-1.03)	*0.63*	0.98(0.89-1.09)	*0.42*	0.94(0.85-1.04)	*0.21*	0.94(0.85-1.04)	*0.20*
III and IV	1,204/2,072	0.97(0.91-1.04)		0.96(0.89-1.03)		0.96(0.90-1.02)		0.96(0.90-1.02)	
BMI, kg/m^2									
< 25	956/2,437	1.04(0.97 -1.11)	*0.48*	1.01(0.94-1.09)	*0.73*	1.01(0.95-1.08)	*0.33*	1.02(0.95-1.09)	*0.34*
25-29.9	1,015/2,406	1.00(0.94-1.06)		1.03(0.96-1.11)		1.02(0.97-1.07)		1.02(0.96-1.07)	
≥ 30	450/951	1.07(0.97 -1.18)		0.99(0.90-1.09)		1.09(0.99-1.21)		1.08(0.98-1.20)	
Physical activity			*0.08*		*0.24*		*0.18*		*0.17*
Inactive	309/875	1.12(0.97-1.29)		1.09(0.95-1.25)		1.11(0.96-1.28)		1.11(0.96-1.28)	
Moderately inactive	758/1,776	0.97(0.91-1.04)		0.97(0.89-1.04)		0.99(0.93-1.04)		0.98(0.93-1.04)	
Moderately active	988/2,250	1.00(0.93-1.07)		0.98(0.91-1.06)		1.01(0.94-1.07)		1.00(0.94-1.07)	
Active	200/515	1.09(0.92-1.29)		1.13(0.93-1.38)		1.02(0.86-1.21)		1.03(0.87-1.23)	
Smoking status									
Never smoker	950/2,371	1.06(0.99-1.14)	*0.87*	1.04(0.97-1.12)	*0.29*	1.08(1.01-1.15)	*0.72*	1.08(1.01-1.15)	*0.74*
Former smoker	820/1,964	0.99(0.93-1.05)		0.98(0.92-1.05)		0.99(0.94-1.05)		0.99(0.94-1.05)	
Current smoker	614/1,386	0.99(0.89-1.09)		1.00(0.90-1.10)		0.96(0.86-1.06)		0.96(0.87-1.07)	
Alcohol intake									
Non-drinkers	175/373	1.07(0.93-1.22)	*0.71*	1.03(0.87-1.21)	*0.63*	1.05(0.92-1.20)	*0.65*	1.06(0.93-1.20)	*0.64*
< sex-specific median[j]	1,118/2,527	1.00(0.94-1.05)		0.99(0.93-1.06)		1.00(0.96-1.05)		1.00(0.95-1.05)	
≥ sex-specific median[j]	1,128/2,901	1.03(0.96-1.11)		1.02(0.95-1.09)		1.02(0.95-1.10)		1.02(0.95-1.10)	
Diabetes[k]									
Yes	256/549	1.00(0.87-1.14)	*0.03*	1.00(0.88-1.14)	*0.02*	0.97(0.85-1.11)	*0.06*	0.98(0.85-1.12)	*0.05*
No	1,813/4,599	1.05(1.00-1.11)		1.04(1.00-1.09)		1.05(1.00-1.10)		1.05(1.00-1.11)	

Abbreviation: CML, Ne-(caroxymethyl)lysine ; CEL, Ne-(1-caroxyethyl)lysine; MGH, Ne-(5-hydro-5-methyl-4-imidazolon-2-yl)-ornithine; BMI, body mass index; dx, diagnosis; SD, standard deviation; yrs, years. [a] SD for CML = 1.01 mg/d;

SD for CEL = 0.73 mg/d; SD for MG-H1 = 8.47 mg/d; and SD for the combined AGEs = 9.83 mg/d. [b] Residuals were computed by a linear regression of the log-transformed intake of AGEs on total energy intake, sex and center. [c] Combined AGEs: CML+CEL+MG-H1. [d] Multivariable cox proportional hazard model, adjusted for sex, age at diagnosis (yrs; continuous), stage (categorical), total energy intake (kcal/d; continuous), year of diagnosis (continuous), location of tumor(categorical), BMI (continuous), smoking status (categorical) and prevalent/incident diabetes (categorical), and stratified by center. In the stratified models, the stratifying variable was not adjusted for. P for interaction is provided unless otherwise indicated. [e] P_{trend}. [f] Missing stage data were imputed using the algorithm described in the Statistics analyses section. [g] Multivariable model including all CRC cases with time interval between age at dx and age at end of follow up, of more than 2,3, and 5 years. [h] Only colon tumors with known locations included. Unspecified and overlapping (n = 254) were excluded. [i] Missing stage variables were not included. [j] Median of alcohol intake for men: 15.7 g/d, for women: 3.7 g/d. [k] Diabetes based on self-report at baseline and ascertainment during follow-up.

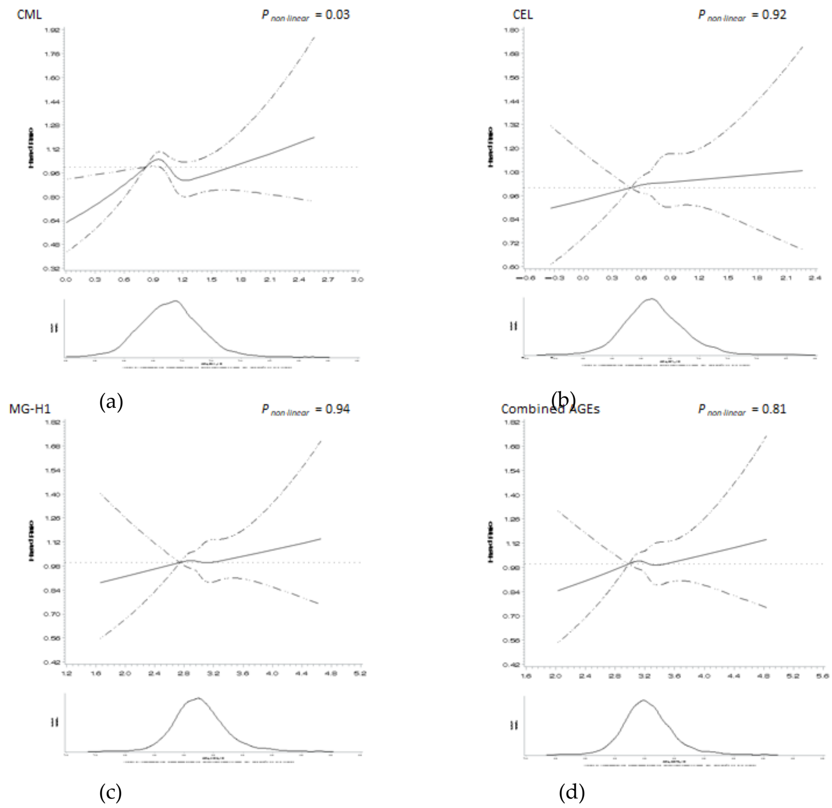

Figure S1. Non-parametrical restricted cubic splines of AGEs with CRC-specific mortality among CRC patients in the EPIC study: (a) splines of CML with CRC-specific mortality; (b) splines of CEL with CRC-specific mortality; (c) splines of MG-H1 with CRC-specific mortality; (d) splines of combined AGEs with CRC-specific mortality.

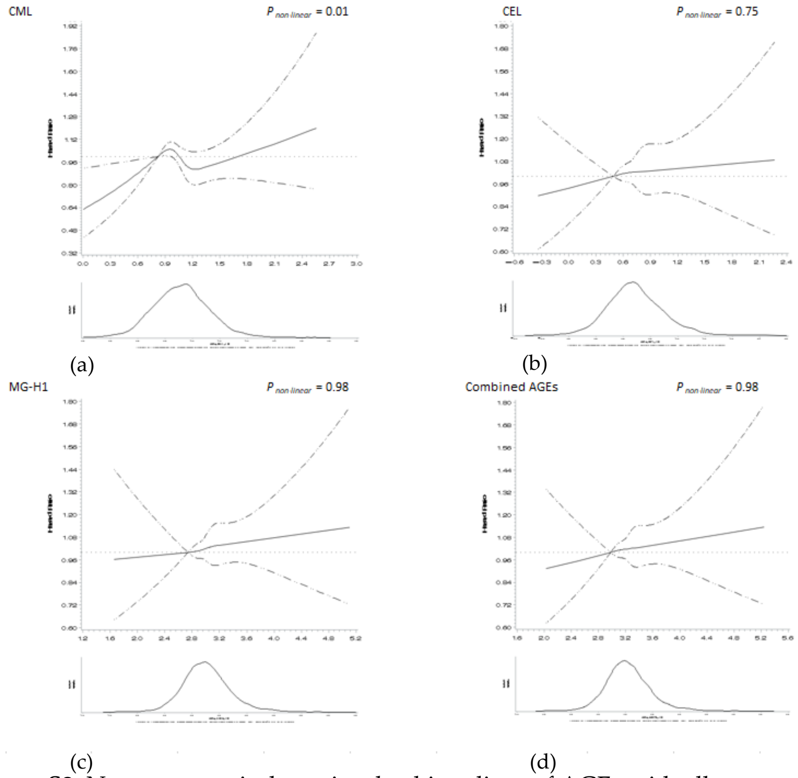

Figure S2. Non-parametrical restricted cubic splines of AGEs with all-cause mortality among CRC patients in the EPIC study: (a) splines of CML with all-cause mortality; (b) splines of CEL with all-cause mortality; (c) splines of MG-H1 with all-cause mortality; (d) splines of combined AGEs with all-cause mortality.
